# Resistance and Resilience of Fish Gut Microbiota to Silver Nanoparticles

**DOI:** 10.1128/mSystems.00630-21

**Published:** 2021-09-14

**Authors:** Pubo Chen, Jie Huang, Liuyu Rao, Wengen Zhu, Yuhe Yu, Fanshu Xiao, Xiaojuan Chen, Huang Yu, Yongjie Wu, Kui Xu, Xiafei Zheng, Ruiwen Hu, Zhili He, Qingyun Yan

**Affiliations:** a Environmental Microbiomics Research Center, School of Environmental Science and Engineering, Southern Marine Science and Engineering Guangdong Laboratory (Zhuhai), Sun Yat-sen University, Guangzhou, China; b Key Laboratory of Aquatic Biodiversity and Conservation of Chinese Academy of Sciences, Institute of Hydrobiology, Chinese Academy of Sciences, Wuhan, China; c Key Laboratory of Ecological Impacts of Hydraulic-Projects and Restoration of Aquatic Ecosystem of Ministry of Water Resources, Institute of Hydroecology, Ministry of Water Resources and Chinese Academy of Sciences, Wuhan, China; d College of Agronomy, Hunan Agricultural University, Changsha, China; University of Hawaii at Manoa

**Keywords:** zebrafish, gut microbiota, resistance, resilience, silver nanoparticles

## Abstract

Understanding mechanisms governing the resistance and resilience of microbial communities is essential for predicting their ecological responses to environmental disturbances. Although we have a good understanding of such issues for soil and lake ecosystems, how ecological resistance and resilience regulate the microbiota in the fish gut ecosystem remains unclear. Using the zebrafish model, we clarified the potential mechanisms governing the gut microbiota after exposure to silver nanoparticles (AgNPs). Here, we explored the ecological resistance and resilience of gut microbiota in zebrafish exposed to different concentrations of AgNPs (i.e., 10, 33 and 100 μg/liter) for 15, 45, 75 days. The high-throughput sequencing analysis of the 16S rRNA gene showed that AgNP exposure significantly reduced the α-diversity of gut microbiota and resulted in obvious dynamics of community composition and structure. However, the rebound of zebrafish gut microbiota was pushed toward an alternative state after 15 days of AgNP exposure. We found that homogeneous selection was a more prevalent contributor in driving gut community recovery after AgNP exposure. The resilience and resistance of gut microbiota responses to AgNP disturbance might be mainly determined by the predominant keystone taxa such as Acinetobacter and *Gemmata*. This study not only expanded our understanding of fish gut microbiota’s responses to pollutants but also provided new insights into maintaining host-microbiome stability during environmental perturbations.

**IMPORTANCE** Understanding the ecological mechanisms governing the resistance and resilience of microbial communities is a key issue to predict their responses to environmental disturbances. Using the zebrafish model, we wanted to clarify the potential mechanisms governing the resistance and resilience of gut microbiota after exposure to silver nanoparticles (AgNPs). We found that AgNP contamination significantly reduced the α-diversity of gut microbiota and resulted in obvious changes in community composition. The resilience and resistance of gut microbiota to AgNPs might be associated with the predominant keystone taxa (e.g., Acinetobacter and *Gemmata*). This study greatly expanded our understanding of how fish gut microbiota responds to environmental perturbations and maintains stability.

## INTRODUCTION

The ecological stability of the microbial community as reflected by resistance and resilience to environmental changes or disturbances is a key issue for understanding ecosystem functions (1, 2). Microorganisms not only are the crucial players in biogeochemical cycling in different ecosystems but are also found to be linked with impairment of ecosystem functions when some microbial taxa are lost (3). Recent studies suggested that microbial communities could also be used for predicting ecosystem responses to global changes (4, 5). However, we still lack a full understanding of how microbial communities respond to ecological disturbances in particular ecosystems or global process models. Therefore, the resistance and resilience of microbial communities during environmental disturbances have received increasing interest during the last 2 decades.

Although the dynamics of taxonomic composition and community diversity are commonly involved in studying microorganisms’ responses to various disturbances, the microbial community assembly processes are also major matters for microbial resistance and resilience (6). Unfortunately, the underlying mechanisms of microbial community assembly and succession subjected to environmental disturbances still remain controversial (1). Generally, ecological patterns of communities can be visualized in four types of processes: selection, drift, speciation, and dispersal (7). It is commonly accepted that selection is a major force shaping microbial communities, which may help microorganisms withstand or adapt to a disturbance (8, 9). Microorganisms are also well-known materials for rapid dispersal in fluid ecosystems (10), and the dispersed members represent an important regional species pool that can colonize in disturbed ecosystems (3). Microbial dispersal can also enhance compositional recovery by reintroducing species that were lost after disturbances. For example, dispersal promoted microbial recovery before a drought episode by reintroducing sensitive taxa at the early stage of rewetting (11). However, the ecological processes governing microbial communities are still not easy to quantify accurately due to some methodological issues (12).

Currently, the potential mechanisms affecting microbial interactions are also used to address the microbial responses to environmental disturbances (13–15). Network analysis has become one of the most common approaches in exploring microbial cooccurrence patterns (16). A disturbance may lead to an increase in the abundance of some taxa as a consequence of the loss of major competitors that could not withstand the disturbance. Moreover, the modularity, average clustering coefficient (avgCC), average path distance (GD), and node centrality of networks are also good properties for characterizing microbial interactions and reflecting microbial resilience (3, 17). For example, a network-based analysis of bacterioplankton communities showed that networks with higher modularity would be more stable than dispersal networks (18). Similarly, the indirect effects of drought could result in lower stability of bacterial networks due to an increase in node centrality but a decrease in modularity (19–21). Thus, network analysis of microbial interactions can also expand our understanding of resistance and resilience during disturbances.

Recently, the extensive use of nanoparticles has resulted in environmental disturbances due to their toxic responses to biological systems (22). Nanoparticles may come from many consumer products, including textiles, cosmetics, plastic, food packaging, and medical appliances (23). Such products are potential sources of environmental release of nanoparticles into aquatic environments, which could pose risks to both natural ecosystems and organisms therein (24). Recent studies on zebrafish (Danio rerio) and common carp (Cyprinus carpio) showed that silver nanoparticles (AgNPs) were ingested and highly accumulated in the intestinal tract to cause gut microbial dysbiosis during acute toxic exposure (25, 26). However, whether gut microbiota affected by AgNP exposure could return to the initial state after environmental recovery is unclear. This study aimed to reveal the ecological resistance and resilience of gut microbiota in fish subjected to disturbance by the AgNPs.

The zebrafish has been an attractive vertebrate model for studying gut microbiota due to its small size, rapid development, optimum breeding, and maintenance conditions (27, 28). It has greatly increased our understanding of the mechanisms governing fish gut microbiota in the past 2 decades (for examples, see reference 29 to 31), which also can be used to investigate the responses of microbial communities to environmental stresses (32). Moreover, the gut ecosystem represents an isolated oceanic “island,” where the microbial community colonizes in the “ocean” of the surrounding environment (33, 34). This study, therefore, used the zebrafish model to clarify the mechanisms of microbial resistance and resilience. We hypothesize that the resistance and resilience of gut microbiota are mainly governed by selecting adaptive taxa and increasing their interactions in response to AgNP disturbances. To test this hypothesis, we used exposures to different concentrations of AgNPs (i.e., 10, 33, and 100 μg/liter) as disturbances to explore their possible effects and the underlying mechanisms maintaining gut microbiota. This study not only increases our understanding of how environmental disturbances affect the fish gut microbiota but also provides novel insights into maintaining stable gut microbiota for host health.

## RESULTS

### Gut microbial diversity and structure in relation to resistance and resilience.

**FIG 1:**
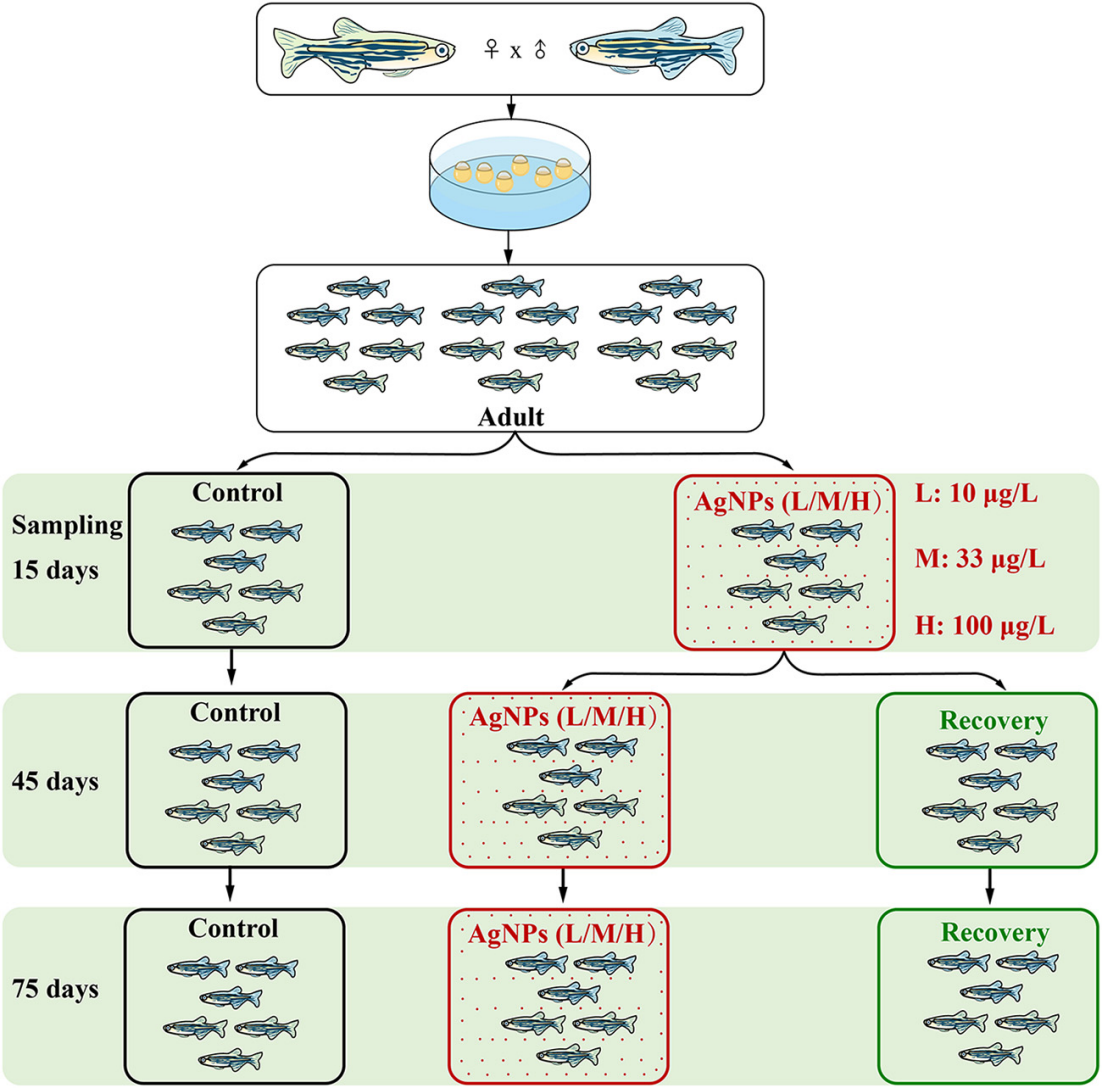
Experimental design. The resilience and resistance of gut microbiota were investigated using the same batch of adult zebrafish, which were subjected to exposure to three concentrations of AgNPs (i.e, 10, 33, and 100 μg/liter) for 15 days. They were then randomly assigned to continued-exposure (red) or recovery (green) groups for 30 and 60 days, respectively. The continued-exposure and recovery groups were used to test microbial resistance and resilience, respectively. The controls (black) without AgNP exposure were also sampled at each sampling time. L, M, and H indicate exposure to low (10 μg/liter), medium (33 μg/liter), and high (100 μg/liter) concentrations of AgNPs, respectively.

**FIG 2:**
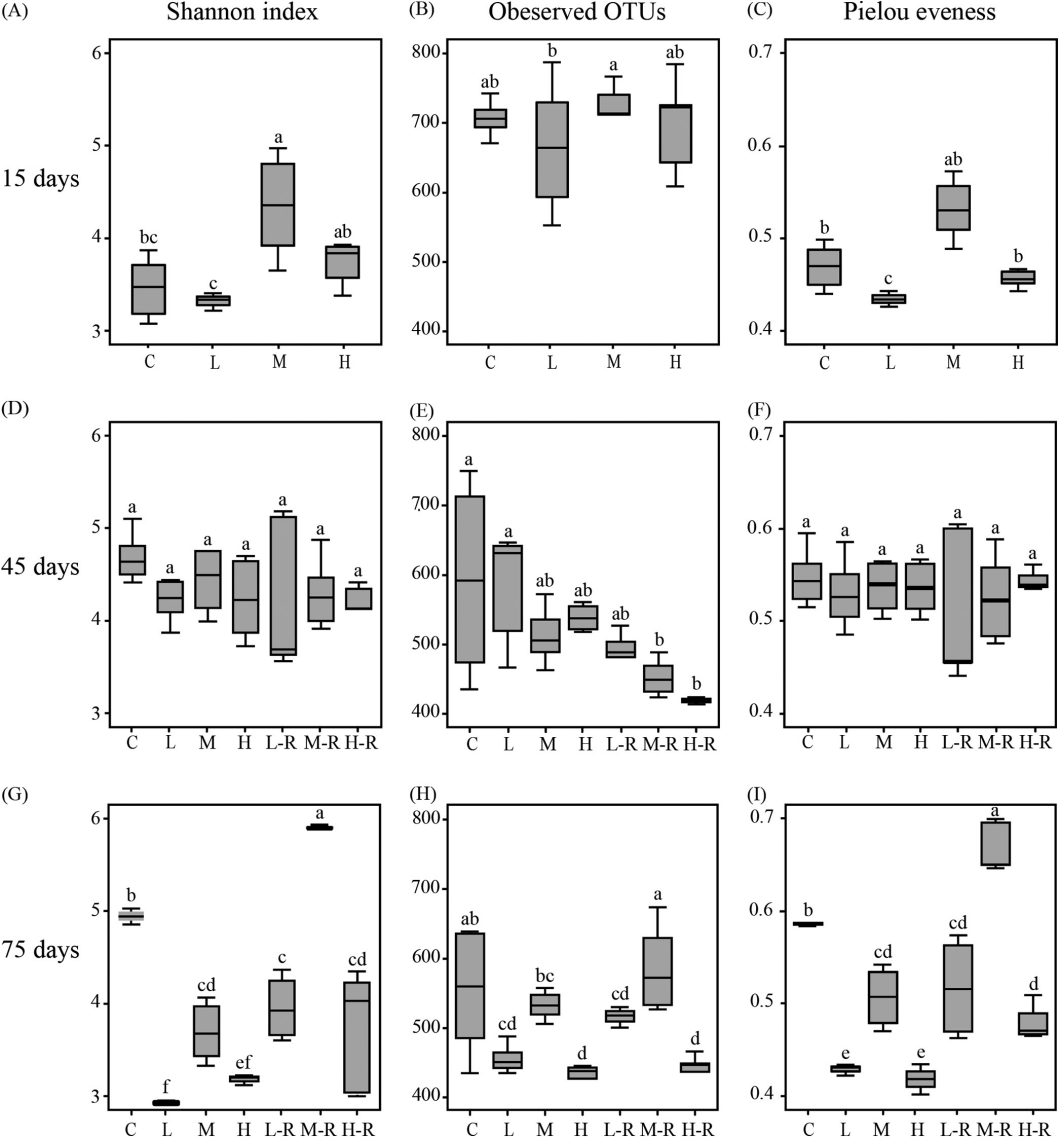
Box plot of Shannon index (A, D, G), observed OTUs (B, E, H), and Pielou evenness index (C, F, I) of gut microbiota in zebrafish after AgNP exposure. Different lowercase letters indicate significant differences (ANOVA, *P < *0.05) among groups. C, control; L, M, and H, exposure to low (10 μg/liter), medium (33 μg/liter), and high (100 μg/liter) concentrations of AgNPs, respectively; L-R, M-R, and H-R, recovery from exposure to low (10 μg/liter), medium (33 μg/liter), and high (100 μg/liter) concentrations of AgNPs, respectively. Data are the mean ± standard error (*n* = 6).

Through sequencing efforts with 14,285 sequences per sample, we classified a total of 6,927 operational taxonomy units (OTUs) (UPARSE, 97% cutoff) for the 105 samples. Zebrafish exposure to AgNPs for 15 days resulted in significant variations in the gut microbiota (Fig. 1). Specifically, the medium concentration exposure (M; 33 μg/liter) showed the highest α-diversity (Fig. 2A to C), while the low concentration exposure (L; 10 μg/liter) showed the lowest (*P < *0.05) after AgNP disturbance. Also, there were significant differences in the Shannon and Pielou evenness indexes between the L and high exposure concentration (H; 100 μg/liter) groups (*P < *0.05). At 45 days, the α-diversity of exposure groups was similar to that of the controls (Fig. 2D to F). In contrast, the Shannon index, observed OTUs, and Pielou evenness index of recovery of groups with low (L-R), medium (M-R), and high (H-R) concentrations of exposure were increasing, as measured at 75 days (Fig. 2G to I). The α-diversity of the M-R group was significantly higher (*P < *0.05) than that of the continued-exposure groups and controls. So, AgNP disturbance significantly decreased the Shannon index, observed OTUs, and Pielou evenness index of gut microbiota at 75 days.

**FIG 3:**
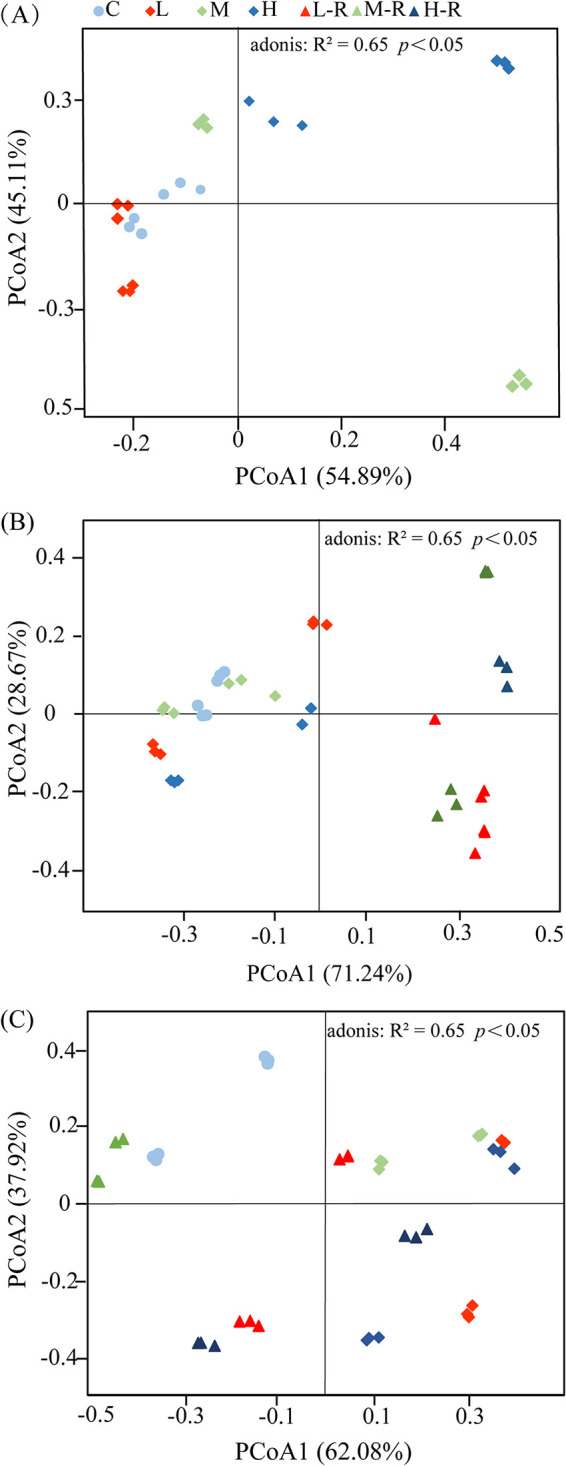
Principal-coordinate analysis (PCoA) of the gut microbiota of zebrafish subjected to AgNP treatments of 15 days (A), 45 days (B), and 75 days (C). C, control; L, M, and H, exposure to low (10 μg/liter), medium (33 μg/liter), and high (100 μg/liter) concentrations of AgNPs, respectively; L-R, M-R, and H-R, recovery from exposure to low (10 μg/liter), medium (33 μg/liter), and high (100 μg/liter) concentrations of AgNPs, respectively.

Principal-coordinate analysis (PCoA) demonstrated that gut microbial structure after 15 days of AgNP disturbance was considerably different from that of controls, but it gradually returned to its normal state after 75 days of recovery (Fig. 3). Although there have some variations among replicates, they do not change the observed trend. With the AgNP concentration increasing, three exposure groups were clearly separated from the controls after 15 days of exposure (Fig. 3A). At 45 days, exposure and recovery groups were almost separated from the controls by the PCoA (Fig. 3B). Surprisingly, given our expectation that AgNP exposure would affect community recovery, a certain similarity of community structure between the recovery groups and controls was detected among gut microbial communities at 75 days (Fig. 3C). So, longer recovery time decreased the difference in microbial structure among groups. These community patterns could be further confirmed by the nonparametric tests (i.e., multiple-response permutation procedure [MRPP], permutational multivariate analysis of variance [PERMANOVA], and analysis of similarity [ANOSIM]) based on the Bray-Curtis distance, which showed significant (*P < *0.05) differences among the gut microbial communities for each pairwise comparison (Table 1).

**TABLE 1:** Dissimilarity tests of gut microbial communities based on three nonparametric tests^*a*^

Sampling time and group comparison	MRPP		ANOSIM		PERMANOVA	
	Delta	*P*	*R*	*P*	*F*	*P*
15 days						
C vs L	0.253	0.001	0.850	0.004	14.153	0.004
C vs M	0.364	0.003	0.495	0.002	6.680	0.002
C vs H	0.340	0.008	0.588	0.003	11.365	0.004
45 days						
C vs L	0.328	0.002	0.909	0.003	18.338	0.004
C vs M	0.283	0.004	0.646	0.001	4.412	0.003
C vs H	0.267	0.005	0.938	0.004	11.380	0.002
C vs L-R	0.305	0.003	0.892	0.002	9.5674	0.007
C vs M-R	0.362	0.004	0.888	0.002	13.091	0.003
C vs H-R	0.234	0.020	1.000	0.0.013	33.933	0.010
75 days						
C vs L	0.352	0.002	1.000	0.003	17.957	0.003
C vs M	0.310	0.006	0.892	0.002	16.502	0.002
C vs H	0.423	0.001	1.000	0.004	11.714	0.003
C vs L-R	0.404	0.005	0.585	0.003	7.777	0.003
C vs M-R	0.361	0.002	0.827	0.003	11.587	0.001
C vs H-R	0.437	0.004	0.580	0.003	7.167	0.004

a C, control; L, M, and H, exposure to treatments of low (10 μg/liter), medium (33 μg/liter), and high (100 μg/liter) concentrations of AgNPs, respectively; L-R, M-R, and H-R, recovery from exposure to low (10 μg/liter), medium (33 μg/liter), and high (100 μg/liter) concentrations of AgNPs, respectively; MRPP, multiple-response permutation procedure; ANOSIM, analysis of similarities; PERMANOVA, permutational multivariate analysis of variance; Delta, a measure of dissimilarity by means of a “delta score”; *R*, the correlation coefficient, where an *R* value near +1 means that there is dissimilarity between the groups, while an *R* value near 0 indicates no significant dissimilarity between the groups; *F*, pseudo-f statistic for testing the null hypothesis.

### Community composition in relation to resistance and resilience.

**FIG 4:**
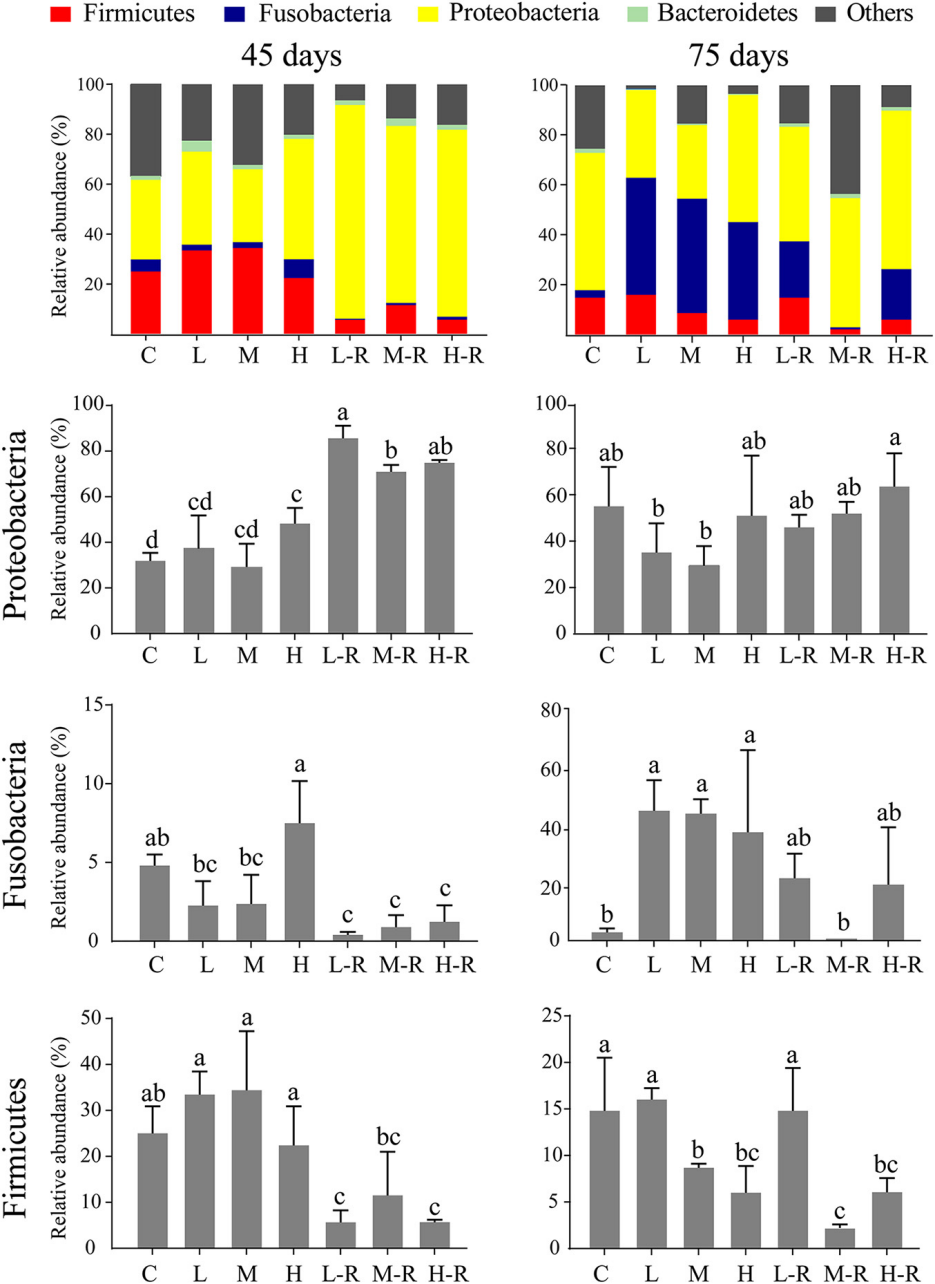
Relative abundances of the dominant phyla of zebrafish gut microbiota at 45 days (left panel) and 75 days (right panel). Different lowercase letters above the bars indicate significant differences among groups (ANOVA, *P < *0.05), whereas the same letter suggests no significant difference. Data are the mean ± standard error (*n* = 6).

Zebrafish gut microbial communities were generally dominated by the phyla *Firmicutes*, *Fusobacteria*, *Proteobacteria*, and *Bacteroidetes* (Fig. 4; see Fig. S1 in the supplemental material). At 45 days, no significant changes were observed between exposure groups and the controls at the phylum level, but *Proteobacteria* detected in the high-concentration exposure group were significantly (*P < *0.05) increased. In contrast, the relative abundance of *Proteobacteria* in all the recovery groups was significantly higher than that in the control and exposure groups (*P < *0.05). In addition, the relative abundance of *Fusobacteria* in the recovery groups ranged from 0.4% to 1.2%, significantly lower than that of the controls. The relative abundance of *Firmicutes* in the recovery groups ranged from 5.6% to 11.5%. However, the relative abundance of *Proteobacteria* at 45 days was negatively correlated with that of *Fusobacteria* and *Firmicutes*, and the relative abundance of *Proteobacteria* and *Fusobacteria* in the recovery groups was not significantly different from that of controls at 75 days.

10.1128/mSystems.00630-21.1 FIG S1 Relative abundance of dominant phyla of zebrafish gut microbiota at 15 days of exposure. Different letters above the bars indicate significant differences among groups (ANOVA, *P < *0.05), whereas the same letter suggests no significant difference. Download FIG S1, TIF file, 0.2 MB. Copyright © 2021 Chen et al. 2021 Chen et al. https://creativecommons.org/licenses/by/4.0/ This content is distributed under the terms of the Creative Commons Attribution 4.0 International license.

**FIG 5:**
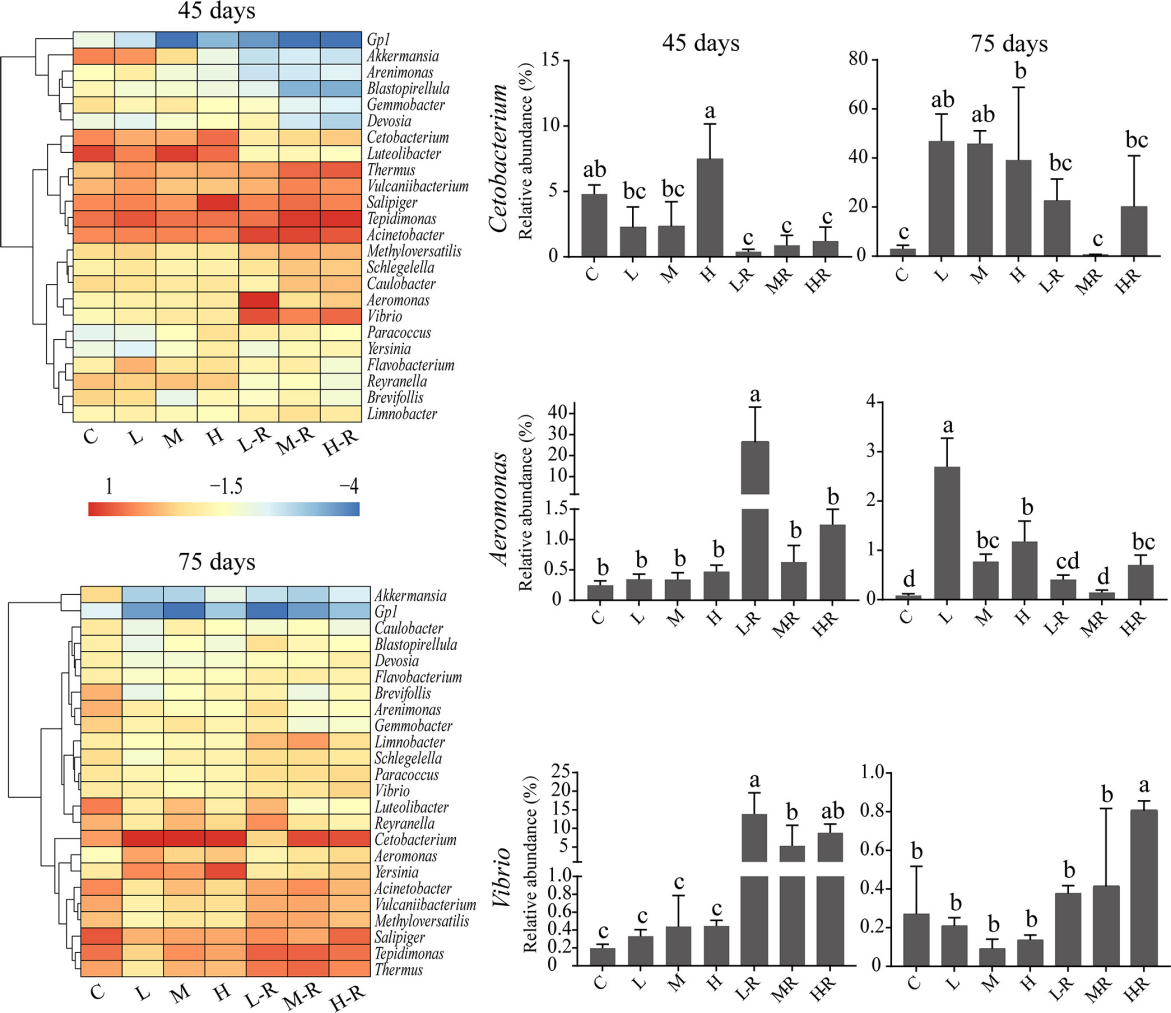
Relative abundances of the dominant genera of zebrafish gut microbiota. Different lowercase letters above the bars indicate significant differences among groups (ANOVA, *P < *0.05), whereas the same letter suggests no significant difference. Data are the mean ± standard error (*n* = 6).

The heatmap with hierarchical clustering of the top 25 genera (Fig. 5) indicated that the most abundant genera across all samples were *Cetobacterium*, *Tepidimonas*, Acinetobacter, *Thermus*, *Vulcaniibacterium*, *Salipiger*, *Luteolibacter*, *Methyloversatilis*, *Paracoccus*, *Vibrio*, and *Aeromonas*. At 45 days, the percentage of *Cetobacterium* ranged from 0.9% to 7.5%, while it ranged from 0.7% to 47.0% at 75 days and showed no significant differences from that of the controls in the recovery groups. The abundance of *Aeromonas* detected in the recovery groups showed significant differences from that in the controls at 45 days, and the L-R and M-R groups showed no significant differences from the controls at 75 days. Similarly, the relative abundance of *Vibrio* in the L-R and M-R groups was not significantly different from that of the controls at 75 days. Among the recovery groups, some genera increased their relative abundances, such as Acinetobacter (from 4.8% to 15.6%), *Tepidimonas* (from 7.2% to 15.4%), and *Thermus* (from 1.2% to 6.7%). In contrast, the abundances of other genera decreased after exposure. For example, *Akkermansia* decreased from 5.2% to 1.6% and *Gemmobacter* decreased from 0.67% to 0.27% in the exposure groups. The relative abundance of *Cetobacterium* in the 75-day exposure groups was positively correlated with that of *Aeromonas*, while the relative abundance of *Aeromonas* in the recovery groups was negatively correlated with that of *Vibrio*. Overall, gut microbial composition dynamics within each treatment during the recovery period revealed only some minor differences.

To better understand the more refined variations among different treatments, we also compared the variations of “core” OTUs (35), which are defined as those detected in over 80% of samples with an abundance of >1% for each sample of the considered groups (Table S1). OTU_1 (*Cetobacterium*), OTU_2 (*Firmicutes*), OTU_3 (*Tepidimonas*), OTU_4 (*Rhodobacteraceae*), and OTU_5 (*Thermus*) were identified as core taxa in all samples. Interestingly, the opportunistic pathogen of OTU_7 (*Citrobacter*) was significantly enriched in the recovery groups at 75 days. At 45 days, 11, 10, and 12 of the core OTUs detected in control, exposure, and recovery groups accounted for 75.8%, 73.8%, and 77.6% of the total abundance in each group. At 75 days, 17, 11, and 14 core OTUs from the control, exposure, and recovery groups accounted for 73.9%, 88.5%, and 69.1% of the total abundances of each group (Fig. S2). Thus, both gut microbial community composition and the abundance of core OTUs in zebrafish gut showed clear dynamics.

10.1128/mSystems.00630-21.2 FIG S2 Core OTUs (shared by more than 80% of the samples and with a relative abundance of >1%) for different treatments. The relative abundances (A) and a Venn diagram showing the number of shared and unique core OTUs (B) in different treatments are shown. Download FIG S2, TIF file, 1.0 MB. Copyright © 2021 Chen et al. 2021 Chen et al. https://creativecommons.org/licenses/by/4.0/ This content is distributed under the terms of the Creative Commons Attribution 4.0 International license.

10.1128/mSystems.00630-21.6 TABLE S1 Taxonomy of each core OTU (shared by >80% samples and with abundances of >1%) at two sampling times (45 days and 75 days). Download Table S1, DOCX file, 0.02 MB. Copyright © 2021 Chen et al. 2021 Chen et al. https://creativecommons.org/licenses/by/4.0/ This content is distributed under the terms of the Creative Commons Attribution 4.0 International license.

### Ecological processes for assessing resistance and resilience.

**FIG 6:**
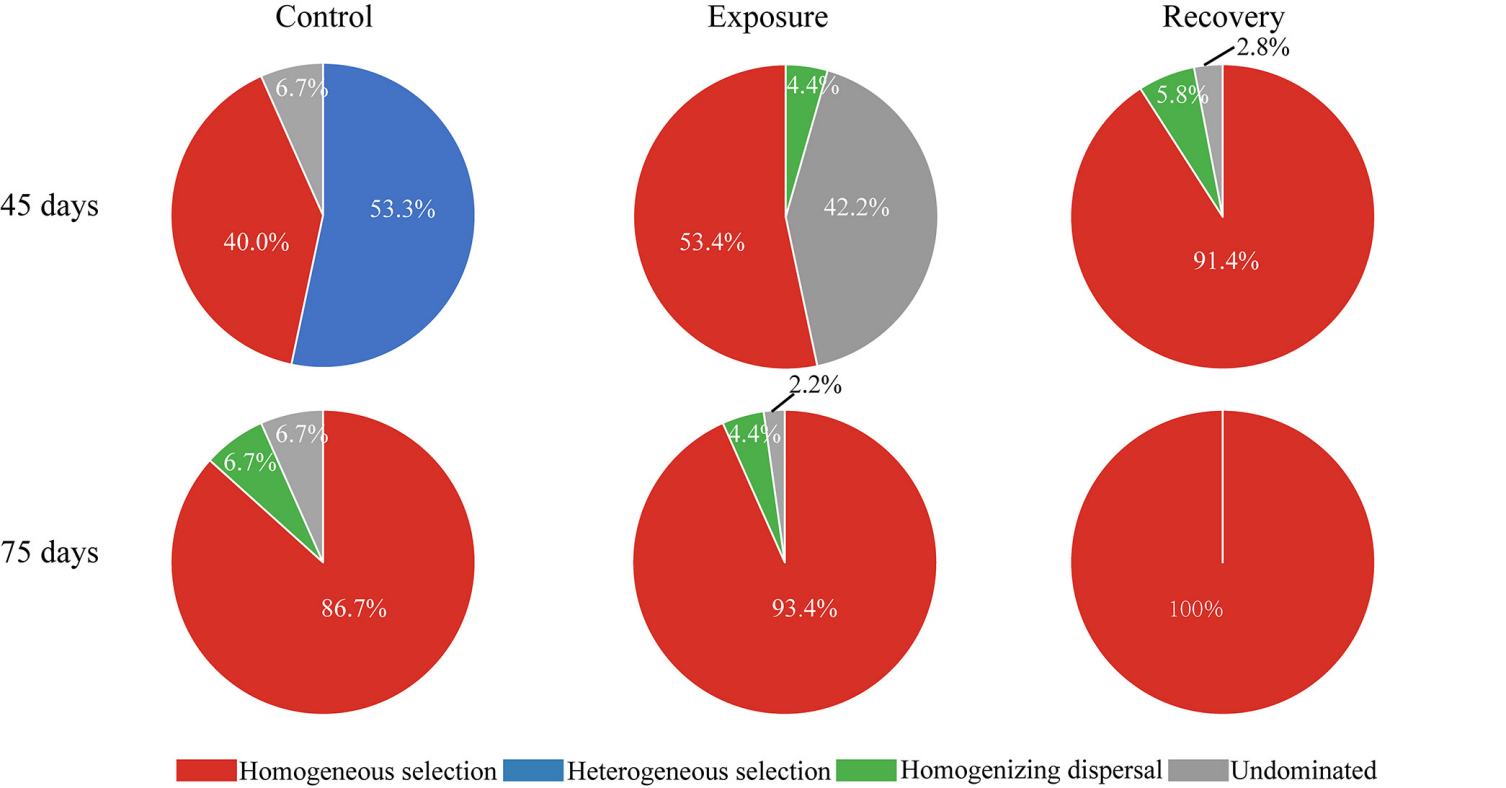
Contribution of ecological processes to the assembly of zebrafish gut microbiota among different treatments.

To clarify the ecological mechanisms underlying the resistance and resilience of microbial community assembly, we further quantified the major ecological processes. We found that the resistance and resilience of gut microbiota were governed by strong deterministic selection processes (Fig. 6). At 45 days, the proportions of deterministic selection (53.4%) and undominated (42.2%) processes were similar in the exposure groups, while the deterministic process was the leading force for microbial assembly in controls (93.4%) and recovery groups (91.4%). At 75 days, deterministic selection was also the major force for microbial assembly in controls (86.7%), exposure groups (93.4%), and recovery groups (100%). Specifically, homogeneous selection, which causes community composition to be similar under consistent environmental conditions, was responsible for 40.0% to 100% of the gut microbial variations and increased with the recovery time. In contrast, there was only weak (4.4%) homogenizing dispersal in the exposure groups, but the contribution of the undominated process was also important (42.2%) throughout the resistance of gut microbiota. Thus, the resilience and resistance of zebrafish gut microbiota appeared to be governed by a deterministic process, especially homogeneous selection in the exposure and recovery groups.

### Networks and interactions to evaluate resistance and resilience.

**FIG 7:**
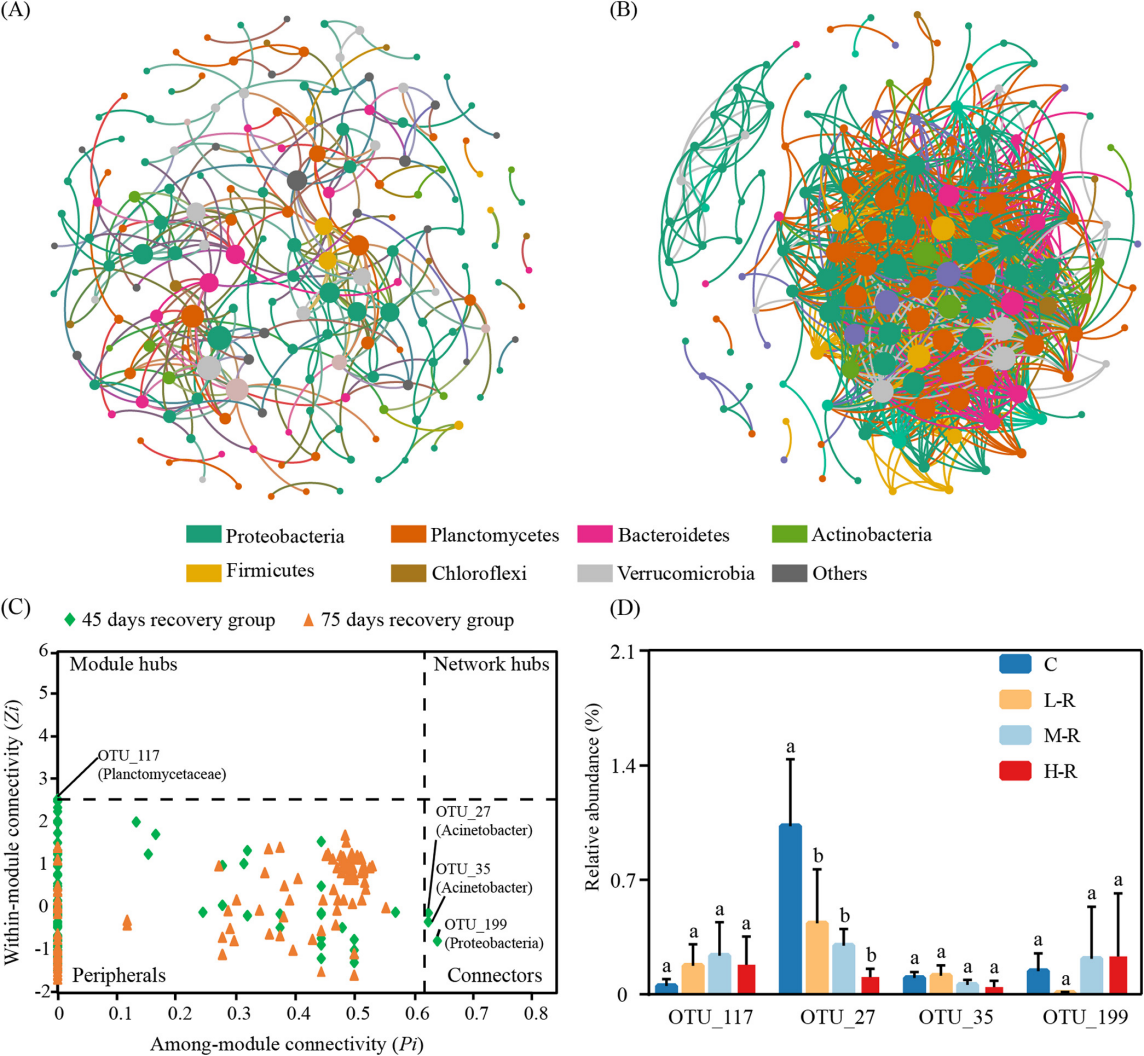
Correlation-based networks of zebrafish gut microbial OTUs in the 45-day (A) and 75-day (B) recovery groups. *Zi*-*Pi* plots show the distribution of OTUs based on their topological roles (C) and the abundance of potential keystone OTUs (D). Each node represents an OTU, and threshold values of *Zi* and *Pi* for categorizing OTUs were 2.5 and 0.62, respectively.

In order to evaluate resistance and resilience with respect to the microbial interactions within zebrafish gut microbiota, correlation-based networks were constructed (Fig. 7A and B; Fig. S3A and B). At 45 days, the community interactions of gut microbiota showed a simple network (average connectivity [avgK], 3.475) but with the highest positive connections (82.0%) in the recovery groups (Table 2). The observed changes were increased with increasing recovery time. The network complexity (a higher average degree representing a greater network complexity) at 75 days for the recovery groups was higher than that at 45 days, but the average path distance (2.161) was slightly reduced. Multiple network topological metrics consistently suggested that networks of treatment groups at 75 days were more complex than those at 45 days. Additionally, we visualized the modules with at least two nodes for further analysis (Fig. S4). The positive links ranged from 62.7% to 93.8%, and the network modularity was >0.174 in all networks (Table 2).

**TABLE 2:** Topological properties of empirical molecular ecological networks in comparison to random networks

Sampling time and group	Total no. of nodes	Empirical networks^*a*^									Random networks^*a*^^,^^*b*^	
		Total no. of links	No. of positive links	avgK	avgCC	GD	Modularity	Nodes with maximum stress centrality	Nodes with maximum eigenvector centrality	Nodes with maximum betweenness	avgCC	GD
45 days												
Control	232	564	408	4.862	0.382	8.274	0.766	OTU_115	OTU_12	OTU_115	0.056 ± 0.009	3.419 ± 0.044
Exposure	257	643	486	5.004	0.283	4.311	0.690	OTU_2	OTU_5	OTU_2	0.691 ± 0.008	1.526 ± 0.002
Recovery	179	311	255	3.475	0.285	6.335	0.740	OTU_59	OTU_3	OTU_59	0.033 ± 0.010	3.856 ± 0.083
75 days												
Control	167	1,236	766	12.026	0.526	3.442	0.479	OTU_109	OTU_87	OTU_109	0.155 ± 0.010	2.748 ± 0.026
Exposure	304	1,828	1,681	14.802	0.471	4.896	0.174	OTU_145	OTU_131 OTU_323	OTU_123	0.227 ± 0.010	2.413 ± 0.026
Recovery	146	1,704	1,584	23.342	0.613	2.161	0.411	OTU_96	OTU_24	OTU_96	0.523 ± 0.015	2.166 ± 0.025

a avgK, average connectivity; avgCC, average clustering coefficient; GD, average path distance.

b Values are the mean ± standard deviation generated from networks randomly rewired 100 times.

10.1128/mSystems.00630-21.3 FIG S3 (A and B) Correlation-based network of zebrafish gut microbial OTUs detected in groups at 45 days (A) and 75 days (B) of exposure. Each node corresponds to an OTU. (C) *Zi-Pi* plots show the distribution of OTUs based on their topological roles in two exposure groups. (D) Abundance of keystone OTUs. Threshold values of *Zi* and *Pi* for categorizing OTUs were 2.5 and 0.62, respectively. Download FIG S3, TIF file, 2.3 MB. Copyright © 2021 Chen et al. 2021 Chen et al. https://creativecommons.org/licenses/by/4.0/ This content is distributed under the terms of the Creative Commons Attribution 4.0 International license.

10.1128/mSystems.00630-21.4 FIG S4 Highly connected modules within zebrafish gut microbial networks for different treatments (control, exposure, and recovery). Colors of nodes indicate different major phyla. Red links indicate positive correlations between two individual nodes, whereas blue links indicate negative correlations between nodes. Download FIG S4, TIF file, 2.9 MB. Copyright © 2021 Chen et al. 2021 Chen et al. https://creativecommons.org/licenses/by/4.0/ This content is distributed under the terms of the Creative Commons Attribution 4.0 International license.

The possible topological roles of nodes in the gut microbiota were determined by their within-module connectivity (*Zi*) and among-module connectivity (*Pi*). There were three connectors (OTU_27, OTU_35, and OTU_199) for the recovery groups at 45 days, but there was no connector for the recovery groups at 75 days (Fig. 7C). Similarly, only one module hub (OTU_117) was detected for the recovery groups at 45 days. The OTU_27 (Acinetobacter) with 0.5% relative abundance showed significant differences from that of the controls at 45 days (Fig. 7D). We also evaluated the contributions of the potential keystone taxa to the exposure groups for resistance. The potential keystone taxa in exposure groups at 45 and 75 days were mainly from *Planctomycetes* (including OTU_54, OTU_73, OTU_124, OTU_235, and OTU_390). Moreover, the relative abundances of OTU_124 (0.6%) (*Planctopirus*), OTU_73 (0.9%) (*Gemmata*), and OTU_54 (0.9%) (*Planctomycetaceae*) were significant different from that of the controls (Fig. S3C and D). Our results showed that AgNP exposure affected the network structure and topological roles of individual OTUs and the potential keystone taxa.

## DISCUSSION

Understanding ecological stability by characterizing community resilience and resistance to disturbances is crucial for predicting microbial responses to environmental changes. However, such knowledge is very limited for animal gut microbiota, which was recently considered to be an extra “organ” of the host (36). We found that the gut microbiota in zebrafish subjected to AgNP exposure was pushed toward an alternative state after recovery of the water environment. Notably, maintenance of the stability of the zebrafish gut microbiota might be attributed mainly to the cooperation of some beneficial microbes, and homogeneous selection was one of the major ecological processes driving gut microbiota recovery after AgNP disturbance. These results supported our hypothesis that gut microbial resilience and resistance were governed by selecting an adaptive taxonomic composition and increasing their microbial interactions in response to environmental disturbances.

Resilience and resistance are inherent properties to characterize the stability of microbial communities, which cannot be independent of the community diversity and composition (37). Generally, higher microbial diversity always shows stronger resistance to continuous disturbances and might be easier to recover after the disturbance is excluded (38). We found that continued AgNP exposure of zebrafish decreased both the diversity and richness of gut microbiota at 15 and 75 days. This finding is similar to that reported in exposure of fathead minnows to a low level of triclosan (39). Moreover, the increasing diversity in medium concentration of exposure and recovery groups indicated that moderate disturbances would destabilize the microbial niches (40). These niches were colonized by new species and thus resulted in increasing diversity (41). We know that the ecotoxicity of nanoparticles could suppress sensitive gut microbes (42) and promote pollutant-tolerant members (e.g., *Proteobacteria*), which may significantly affect the composition of gut microbiota in zebrafish exposed to AgNPs. This was confirmed by the community patterns at 15 and 75 days, as visualized in PCoA ordination. The disturbed microbial community could, after a certain time of environmental recovery, rebound to a state comparable to that before disturbance but not to a complete recovery. This result was identical to that of human microbiomes perturbed with antibiotic exposure (43). On the other hand, a convergent trend of microbial patterns between the exposure groups and controls at 45 days indicated that the gut microbiota also could resist some of the AgNP disturbance.

The variations of specific microbial taxa in the zebrafish gut may also affect gut stability after AgNP disturbance. For example, the rapid growth of *Proteobacteria*, which is considered a potential diagnostic signature of dysbiosis and disease risk, may induce the gut microbiota to an unstable state (44, 45). The higher abundance of *Proteobacteria* was also a typical feature of zebrafish with inflammatory bowel disease induced by environmental pollutants (46). In contrast, the cooperation of beneficial microbes such as *Fusobacteria*, *Bacteroidetes*, and *Firmicutes* could result in gut stability by producing essential vitamins to assist in detoxification (47). The significant differences of particular genera between the recovery groups and controls also suggested an incomplete recovery. For example, there was a significant increase of fish pathogens (e.g., *Vibrio* and *Aeromonas*) in the recovery groups. The appearance of specific core OTUs in the gut may be partly due to a different selective pressure within the host gut habitat (48). AgNP disturbance can facilitate the proliferation of opportunistic pathogens (e.g., core OTU_7 [*Citrobacter*]), consistent with other studies of fish microbiota recovery (49). Thus, changes in abundance of microbial taxa at different levels all represented direct resistant and resilient responses to AgNP exposure.

Our findings also revealed the roles of ecological processes affecting the resistance and resilience of gut microbiota to AgNP exposure. Dispersal and colonization are two stochastic forces that lead to unpredictable variability in community composition (50–52). This study indicated that homogenizing dispersal was important only in exposure groups, with the explanation that the microbial diversity showed no significant decrease after 45 days of AgNP exposure. We found that the deterministic process of homogeneous selection governed over 53% of community turnover. That means that the responses of gut microbiota to AgNP disturbance could be explained by the insurance hypothesis, as the taxonomic composition is resilient to disturbances by increasing abundance and compensating for functions previously carried by sensitive taxa. This finding is consistent with a previous study that suggested that the relative importance of selection contributed to microbial resilience by accelerating the adaptation of communities (9). This may be partly due to the rapid growth of *Cetobacterium*, as it can quickly adapt to suitable host conditions (53). Of course, some other processes, such as microbial interactions and active dispersal currently classified as the undominated process, may also facilitate the resilience and resistance of fish gut microbiota.

However, neither composition nor ecological processes can demonstrate the microbial interactions to address the resistance and resilience of gut microbiota. Network analyses were applied to illustrate potential microbial community interactions and explore the resistance of native taxa (54, 55). The higher avgCC detected herein revealed a highly complex and modular microbiome, which might resist environmental disturbances more efficiently. A recent study found that complex microbial networks could increase intestinal microbial stability in Atlantic salmon after exposure to antibiotics (56). Therefore, the microbial resilience and resistance might increase the stability of the cooccurrence network of microbial communities in zebrafish subjected to AgNP exposure. Nevertheless, the stability of the network did not recover to its original level in the recovery group. Moreover, increasing positive interactions were observed in the AgNP exposure groups, indicating an enhancement of community stability. Evidence has suggested that a moderate disturbance would enhance the complexity of microbial interactions (57). In mutualistic ecosystems, beneficial microbes have evolved a distinct defense strategy to protect against pathogens and maintain community stability (58, 59). One previous study suggested that increasing cooperative relationships within microbe interactions may help them be more tolerant of disturbances (41). Collectively, cooperative relationships and complex microbial interactions of zebrafish gut microbiota represented good resistance and resilience to AgNP disturbance.

Also, highly connected taxa have been proposed to be potential keystone taxa, which may play a vital role in restoring a dysbiosis community to a predisturbance composition (60). The concentrated keystone taxa suggest that they may maintain the stability of the gut microbiota at 45 days. We found that the keystone taxa occupied a great proportional influence in the intestinal microbiota, and their absence may lead to network fragmentation in the treatment groups (61). For example, the representative keystone genus in the recovery process is Acinetobacter, which might positively affect the digestive processes of fish because of enzyme production (62). Similarly, the keystone genus of *Gemmata* has been identified to have a versatile hydrolytic capability and a high ability to decompose organic matters by relieving the digestive pressure of intestines to enhance the process of resistance (63). Furthermore, due to the lack of peptidoglycan in the cell wall of *Planctomycetes*, they are able to resist harsh environments and become the keystone of the process of resistance (64). As a result, our attempts exemplified that the stable keystone taxa in the cooccurrence patterns of gut bacterial assembly contributed to gut microbial resilience and resistance.

In conclusion, the AgNP exposure of zebrafish resulted in gut microbiota changes and reduced its α-diversity. We also found that AgNP exposure pushed gut microbial communities toward an alternative state that could facilitate the proliferation of opportunistic pathogens (e.g., *Citrobacter*). The resilience of zebrafish gut microbiota responses to AgNP disturbances might be correlated with selecting an adaptive taxonomic composition and increasing key taxonomic interactions. Additionally, our results indicated that the microbial community resistance and recovery were governed mainly by a deterministic selection process. This ecological resilience and resistance of the host-associated microbiome increased our understanding of environmental disturbance implications for fish gut ecology.

## MATERIALS AND METHODS

### AgNP characterization and solution preparation.

AgNPs (99%; CAS no. 576832-5G) used in this experiment were purchased from Sigma-Aldrich (St. Louis, MO, USA). Although the diameter of the AgNPs was suggested to be approximately 100 nm, we measured their size distribution by dynamic light scattering (DLS) using a Malvern Zetasizer Nano ZS in ultrapure water (100 μg/liter). The size and morphology of AgNPs were characterized by transmission electron microscopy (TEM) and X-ray diffraction (XRD) (see Fig. S5 in the supplemental material). For the stock preparation, AgNP powder was suspended in ultrapure water (Millipore, Billerica, MA) at a concentration of 100 mg/liter. To maintain a stable AgNP concentration, the initial suspension was sonicated (50 W/liter, 40 kHz) for 50 min at 60°C. The stock solution was diluted before use with fresh charcoal-filtered water and resonicated (50 W/liter, 40 kHz). The AgNP stock solutions were then prepared every 48 h to maintain relatively consistent exposure levels. Finally, half of the exposure water was refreshed daily, and the entire exposure water was refreshed weekly to keep consistent concentrations of AgNPs in each tank.

10.1128/mSystems.00630-21.5 FIG S5 Characteristics of AgNPs. (A) AgNP image taken by a transmission electron microscope (TEM). (B) Particle size dispersion of the AgNPs measured by dynamic light scattering (DLS). (C) X-ray diffraction (XRD) patterns of AgNPs. FIG S5, TIF file, 1.4 MB Copyright © 2021 Chen et al. 2021 Chen et al. https://creativecommons.org/licenses/by/4.0/ This content is distributed under the terms of the Creative Commons Attribution 4.0 International license.

### Experimental setup and zebrafish husbandry.

To investigate the ecological resistance and resilience of the gut microbial community, we monitored the succession of gut microbiota in experimental zebrafish (wild type) subjected to the disturbance of AgNP exposure. Adult zebrafish (AB strain) were reared using glass tanks and fed newly hatched brine shrimp (Artemia nauplii) twice daily. Specifically, fully aerated tap water was used during the experiment, and relatively stable conditions (28°C ± 0.5°C and a 14-h light/10-h dark cycle) were applied to avoid additional environmental perturbations. The adult zebrafish were exposed to three different concentrations of AgNPs (i.e., 10, 33, and 100 μg/liter), accompanied by a control group without AgNP exposure. After the zebrafish were exposed to different concentrations of AgNPs for 15 days, they were randomly assigned to continued exposure (Fig. 1, indicated by red) and recovery (Fig. 1, indicated by green) groups. The experiment was then extended an additional 2 months after 15 days of exposure to test the resistance and resilience of the gut microbiota.

### Sampling procedures and DNA extraction.

At each sampling time point (i.e., 15, 45, and 75 days), three female and three male zebrafish were randomly collected from each group (Fig. 1) for gut microbial analysis by sequencing the 16S rRNA gene. However, sex factor was not considered in further analysis due to no statistical differences among females and males (data no shown). The whole intestine of each zebrafish was immediately removed aseptically as previously described (31), and each intestine was regarded as a single sample and stored in a sterile 1.5-ml tube at −80°C until DNA extraction. In total, 105 zebrafish gut samples were obtained for the following DNA extraction. All protocols involved in the animal experiments were approved by the Institutional Animal Care and Use Committee of the Institute of Hydrobiology, Chinese Academy of Sciences (approval identifier Keshuizhuan 08529).

Genomic DNA was extracted using the PowerFecal (gut samples) DNA isolation kit (Mo Bio, CA, USA) in accordance with the manufacturer’s instructions. The concentrations and quality of extracted DNA were determined using a NanoDrop One spectrophotometer (Thermo Fisher Scientific, MA, USA), and all samples were diluted to the same concentration (10 ng/μl) for subsequent PCR amplification.

### Sequencing analysis of the 16S rRNA gene.

The V4 region of the 16S rRNA gene was amplified by the primer set 515F (5′-GTGCCAGCMGCCGCGGTAA-3′) and 806R (5′-GGACTACHVGGGTWTCTAAT-3′). Each sample was amplified in a reaction volume of 50 μl containing 1× Premix *Taq* DNA polymerase (buffer, deoxynucleoside triphosphate [dNTP], and *Taq* were included), 0.2 mM each primer, and 50 ng genomic DNA by using the following procedures: predenaturation for 5 min at 95°C and then 30 cycles of 30 s at 95°C, 30 s at 52°C, and 30 s at 72°C, followed by a postextension of 10 min at 72°C. Negative-control experiments were always performed to ensure that no contamination had occurred. PCR products were visualized using 1% agarose gels stained with ethidium bromide. After all samples were successfully amplified, the PCR products of each sample were quantified and equally combined. The target band visualized by 2.0% agarose gel was excised and purified with a QIAquick gel extraction kit (Qiagen, CA, USA). After requantification of the concentration of the purified DNA, it was subjected to library construction. The constructed amplicon library was finally sequenced by the Illumina HiSeq 2500 platform (Illumina, CA, USA) at Guangdong Magigene Biotechnology Co., Ltd., with a 2 × 250 bp kit.

Quality filtering and processing of sequence reads were conducted using the publicly available Galaxy pipeline (http://mem.rcees.ac.cn:8080/) as described previously (65). In brief, overlapped paired-end sequences were first assembled using FLASH (66), and poorly overlapped and low-quality sequences such as those with a length of <140 bp and a moving-window (5 bp) quality score of <20 were removed before downstream analysis. After removal of chimeras, UPARSE was used to classify high-quality sequences into operational taxonomy units (OTUs) at a cutoff of 97% identity. To make the sequencing depth the same for all samples, all 105 samples were rarefied to 14,825 reads per sample for subsequent analyses.

### Ecological process analysis.

To quantify the ecological processes governing the gut microbial community, we calculated the major ecological processes as previously described (30, 65). In this framework, the variation or turnover of both phylogenetic diversity and taxonomic diversity was first measured with the null model-based phylogenetic and taxonomic β-diversity metrics. The β-nearest taxon indices (β-NTI) and Raup-Crick index (RC_Bray_) were then used to quantify the ecological processes that influence gut microbial community composition on a spatiotemporal scale. The community turnover is governed by heterogeneous (β-NTI > 2) or homogeneous (β-NTI less than −2) selection. Pairwise comparisons with a |β-NTI| of <2 were further subjected to RC_Bray_: the fraction of pairwise comparisons with a |β-NTI| of <2 and an RC_Bray_ of less than −0.95 estimated the homogenizing dispersal influence; the fraction of pairwise comparisons with a |β-NTI| of <2 and an RC_Bray_ of >0.95 estimated the dispersal limitation influence; and the remaining fraction of pairwise comparisons with a |β-NTI| of <2 and an |RC_Bray_| of <0.95 represented the component of compositional turnover undominated by any process mentioned above (67).

### Molecular ecological network construction.

To reveal the gut microbial interactions in response to AgNP disturbance, we constructed networks with the publicly available Molecular Ecological Network Analysis Pipeline (MENA; http://ieg2.ou.edu/MENA/) (17) based on the OTU relative abundances. Covariations were measured across 6 to 18 biological replicates for each network, and only OTUs detected in more than two-thirds of the samples of each group were kept in network construction. Random matrix theory (RMT) was used to automatically identify the appropriate similarity threshold (*St*) prior to network construction (68), but all the networks were constructed using the same *St* (i.e., 0.92). Two topological parameters estimated the roles of individual nodes (OTUs) in the network: the within-module connectivity, *Zi*, which quantified to what extent a node connected to other nodes in its module, and the among-module connectivity, *Pi*, which quantified how well the node connected to different modules. The nodes with a high value of either *Zi* or *Pi* were defined as potential keystone taxa, including module hubs (*Zi *> 2.5, *Pi *≤ 0.62; critical to the module coherence), connectors (*Zi *≤ 2.5, *Pi *> 0.62; connect modules together and important to network coherence), and network hubs (*Zi *> 2.5, *Pi *> 0.62; vital to both the network and its own module coherence). The networks were graphed using Gephi 0.9.2 and Cytoscape 3.8.2.

### Statistical analysis.

The comparisons of α- and β-diversities were used to indicate changes in the gut microbiota in response to AgNP disturbances. The indices of Shannon, Pielou’s evenness, and observed OTUs were calculated using the VEGAN package (v.2.3.5) in R (v.3.4.4). In order to elucidate whole patterns of microbial communities, Bray-Curtis distance was used to reveal community structure based on principal-coordinate analysis (PCoA). A heatmap was constructed between the relative abundances of dominant OTUs using the AUTOMAP package. The multiple-response permutation procedure (MRPP), permutational multivariate analysis of variance (PERMANOVA), and analysis of similarity (ANOSIM) tests were performed to test the community dissimilarity by the VEGAN package in R (version 3.4.4) (69). Significance tests were performed using one-way analysis of variance (ANOVA) with Tukey’s test by using GraphPad Prism 8 (GraphPad Software, Inc., San Diego, CA, USA). A *P* value of <0.05 indicated significant differences, and all values are presented as the mean ± standard error of the replicates in each group.

### Data availability.

The raw sequencing data are available at the Sequence Read Archive (SRA) of the National Center for Biotechnology Information (NCBI) under accession number PRJNA668536.
