# Supplementary figures and images for: Resistance and Resilience of Fish Gut Microbiota to Silver Nanoparticles
Source: mSystems. 2021 Sep 14;6(5):e00630-21. doi: 10.1128/mSystems.00630-21 (PMC8547456; doi:10.1128/mSystems.00630-21)

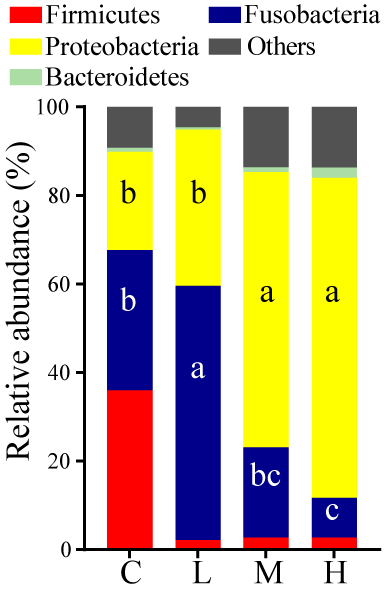

Supplement: FIG S1 [file msystems.00630-21-sf001.tif]

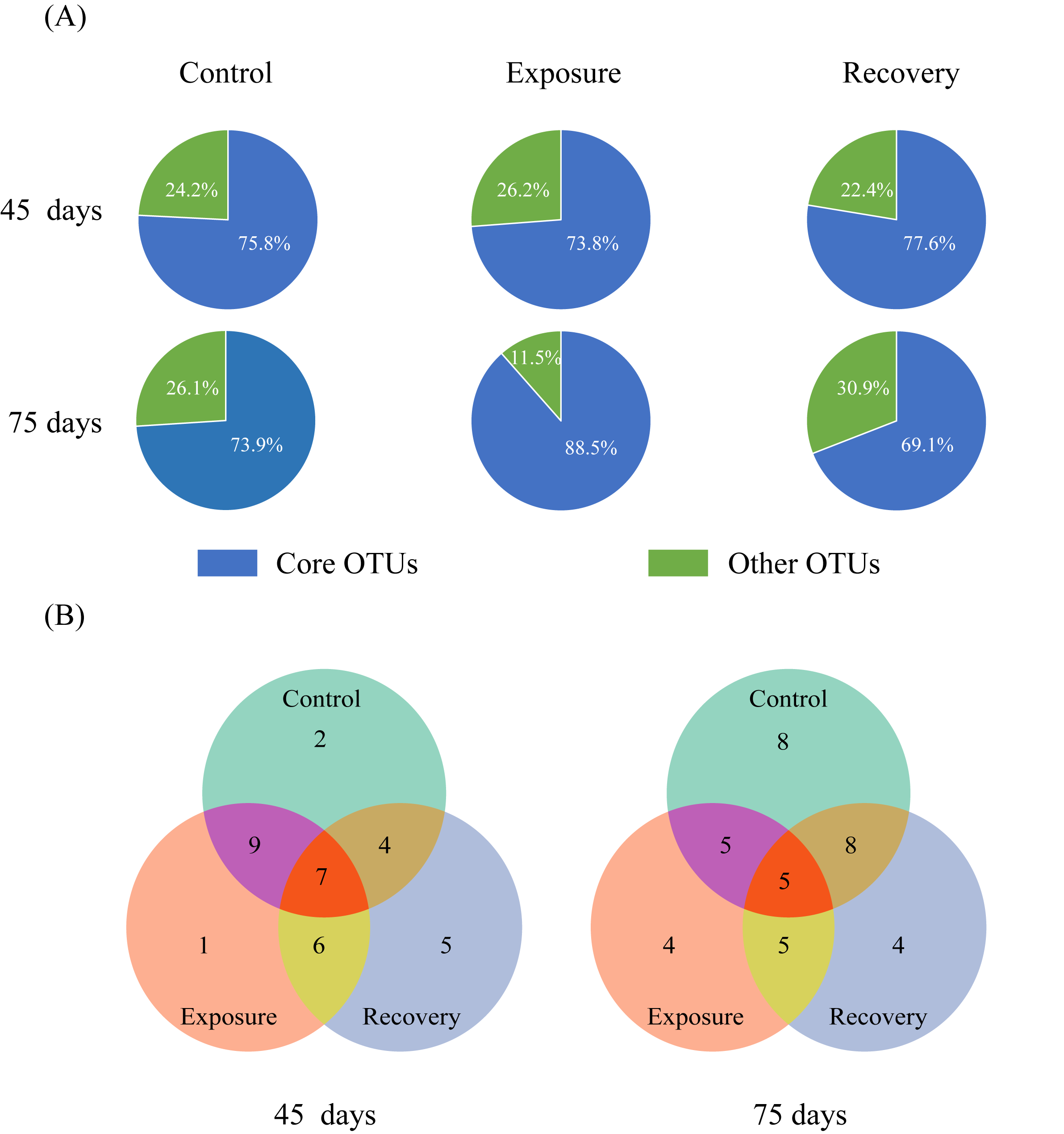

Supplement: FIG S2 [file msystems.00630-21-sf002.tif]

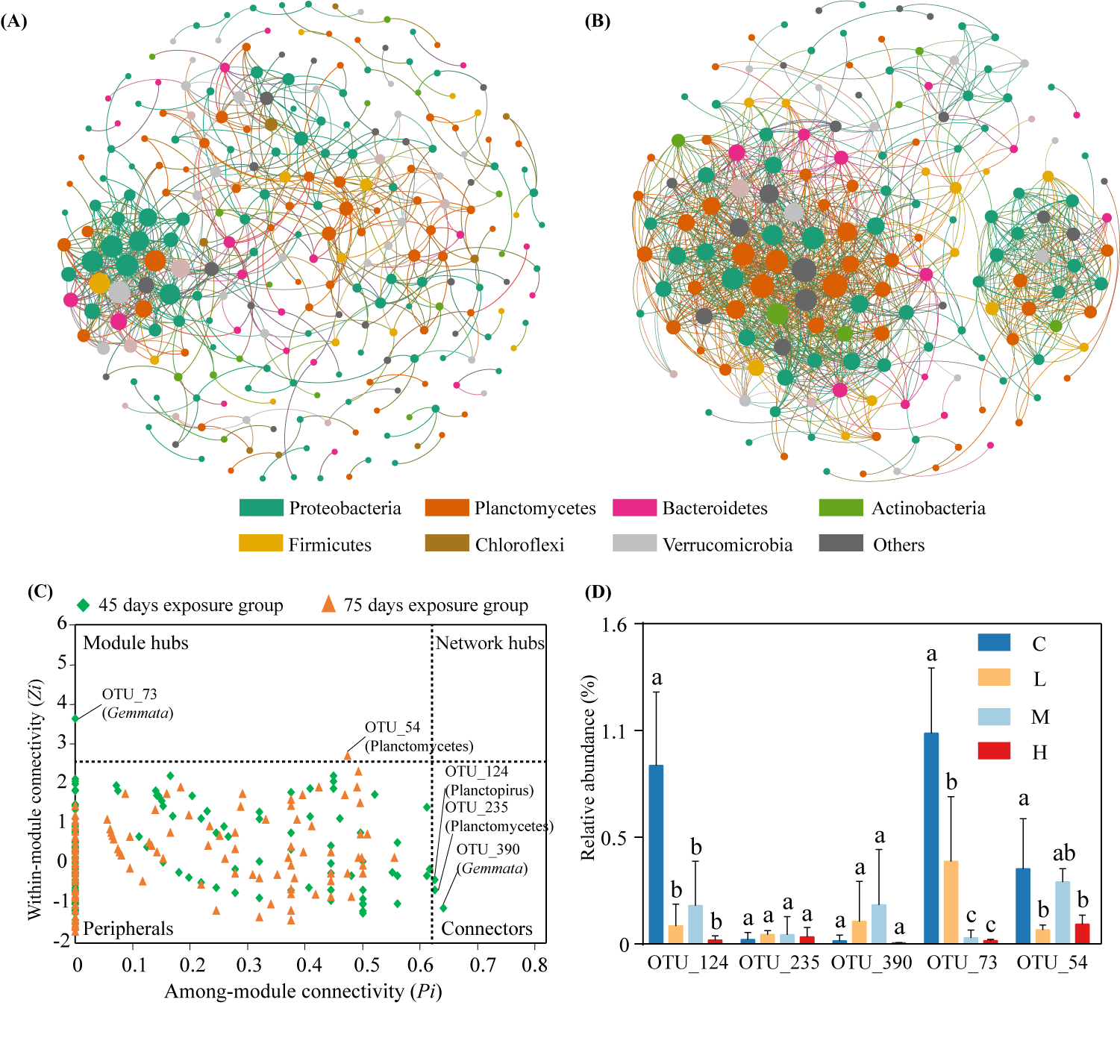

Supplement: FIG S3 [file msystems.00630-21-sf003.tif]

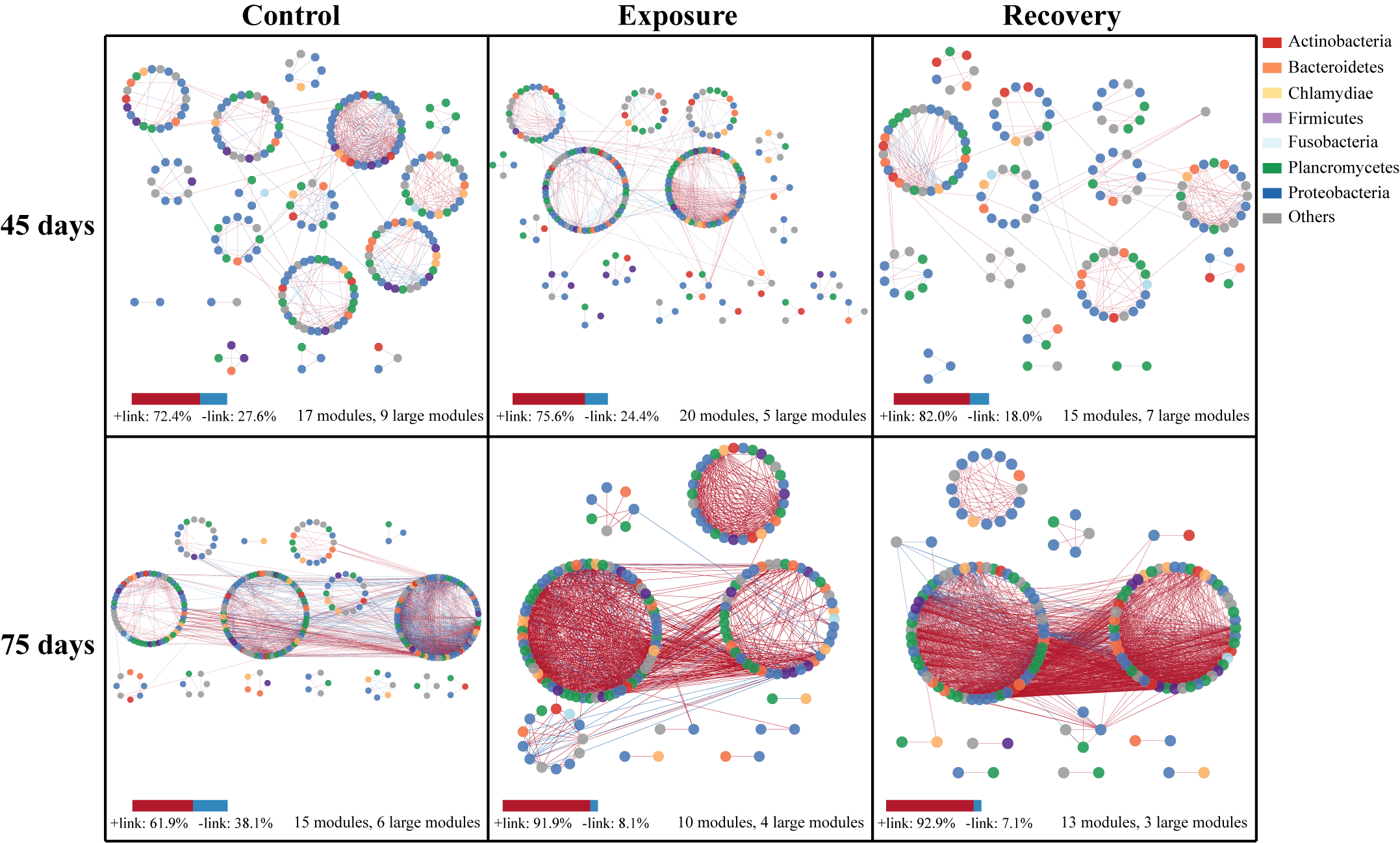

Supplement: FIG S4 [file msystems.00630-21-sf004.tif]

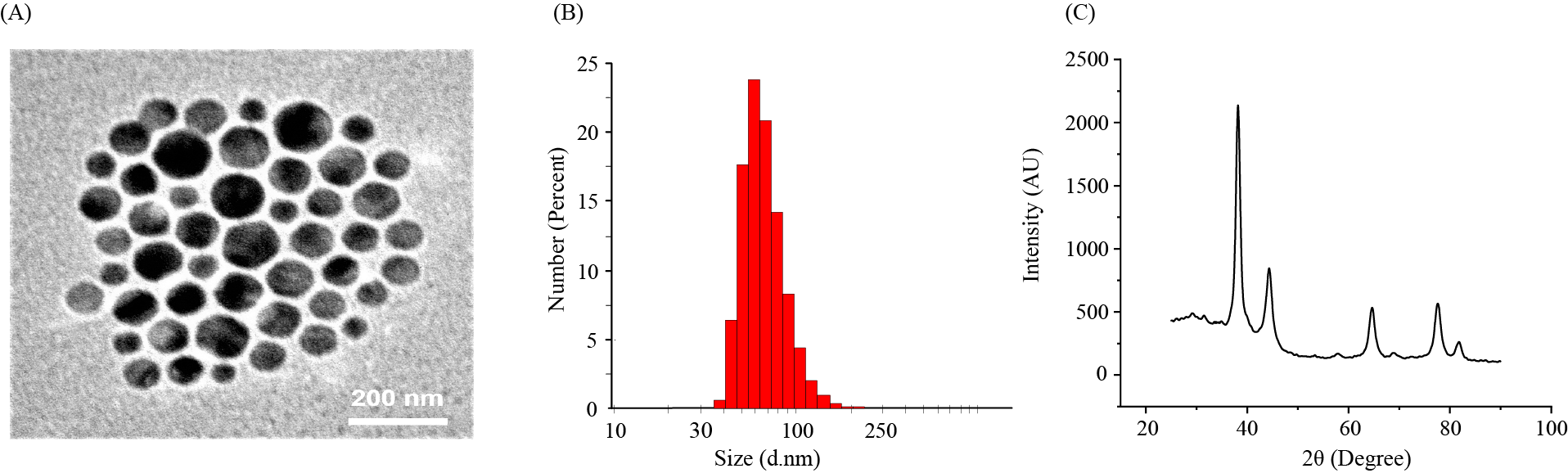

Supplement: FIG S5 [file msystems.00630-21-sf005.tif]
