# Supplementary material for: Resistance and Resilience of Fish Gut Microbiota to Silver Nanoparticles
Source: mSystems. 2021 Sep 14;6(5):e00630-21. doi: 10.1128/mSystems.00630-21 (PMC8547456; doi:10.1128/mSystems.00630-21)
Supplement: TABLE S1 [file msystems.00630-21-st001.docx]

|  | **OTU ID** | **Phylum** | **Class** | **Order** | **Family** | **Genus** |
| --- | --- | --- | --- | --- | --- | --- |
| **45 days** | **OTU_1** | Fusobacteria | Fusobacteriia | Fusobacteriales | Fusobacteriaceae | *Cetobacterium* |
|  | **OTU_2** | Firmicutes | Unclassified | Unclassified | Unclassified | Unclassified |
|  | **OTU_3** | Proteobacteria | Betaproteobacteria | Burkholderiales | Burkholderiales_incertae_sedis | *Tepidimonas* |
|  | **OTU_4** | Proteobacteria | Alphaproteobacteria | Rhodobacterales | Rhodobacteraceae | Unclassified |
|  | **OTU_5** | Deinococcus-Thermus | Deinococci | Thermales | Thermaceae | *Thermus* |
|  | **OTU_6** | Verrucomicrobia | Verrucomicrobiae | Verrucomicrobiales | Verrucomicrobiaceae | *Luteolibacter* |
|  | **OTU_8** | Proteobacteria | Gammaproteobacteria | Pseudomonadales | Moraxellaceae | *Acinetobacter* |
|  | **OTU_10** | Proteobacteria | Gammaproteobacteria | Aeromonadales | Aeromonadaceae | *Aeromonas* |
|  | **OTU_11** | Proteobacteria | Gammaproteobacteria | Xanthomonadales | Xanthomonadaceae | *Vulcaniibacterium* |
|  | **OTU_12** | Proteobacteria | Gammaproteobacteria | Vibrionales | Vibrionaceae | *Vibrio* |
|  | **OTU_13** | Proteobacteria | Betaproteobacteria | Rhodocyclales | Rhodocyclaceae | *Methyloversatilis* |
|  | **OTU_23** | Verrucomicrobia | Verrucomicrobiae | Verrucomicrobiales | Verrucomicrobiaceae | *Akkermansia* |
|  | **OTU_25** | Planctomycetes | Planctomycetia | Planctomycetales | Planctomycetaceae | Unclassified |
|  | **OTU_27** | Proteobacteria | Gammaproteobacteria | Pseudomonadales | Moraxellaceae | *Acinetobacter* |
|  | **OTU_31** | Proteobacteria | Betaproteobacteria | Burkholderiales | Comamonadaceae | *Schlegelella* |
|  | **OTU_36** | Bacteroidetes | Flavobacteriia | Flavobacteriales | Flavobacteriaceae | *Flavobacterium* |
|  | **OTU_45** | Proteobacteria | Betaproteobacteria | Unclassified | Unclassified | Unclassified |
| **75 days** | **OTU_1, OTU_2, OTU_3, OTU_4, OTU_5, OTU_6, OTU_8, OTU_10, OTU_11, OTU_13** | | | | | |
|  | **OTU_7** | Proteobacteria | Gammaproteobacteria | Enterobacteriales | Enterobacteriaceae | *Citrobacter* |
|  | **OTU_9** | Proteobacteria | Gammaproteobacteria | Enterobacteriales | Enterobacteriaceae | *Yersinia* |
|  | **OTU_14** | Unclassified | Unclassified | Unclassified | Unclassified | Unclassified |
|  | **OTU_15** | Firmicutes | Clostridia | Clostridiales | Peptostreptococcaceae | *Clostridium XI* |
|  | **OTU_16** | Actinobacteria | Actinobacteria | Actinomycetales | Nocardiaceae | *Nocardia* |
|  | **OTU_17** | Proteobacteria | Gammaproteobacteria | Xanthomonadales | Xanthomonadaceae | *Arenimonas* |
|  | **OTU_18** | Planctomycetes | Planctomycetia | Planctomycetales | Planctomycetaceae | Unclassified |
|  | **OTU_20** | Proteobacteria | Betaproteobacteria | Neisseriales | Neisseriaceae | *Deefgea* |
|  | **OTU_28** | Proteobacteria | Alphaproteobacteria | Caulobacterales | Caulobacteraceae | *Caulobacter* |
|  | **OTU_24** | Proteobacteria | Alphaproteobacteria | Rhodospirillales | Unclassified | *Reyranella* |
|  | **OTU_26** | Proteobacteria | Betaproteobacteria | Burkholderiales | Burkholderiaceae | *Limnobacter* |
|  | **OTU_33** | Planctomycetes | Planctomycetia | Planctomycetales | Planctomycetaceae | *Zavarzinella* |
|  | **OTU_38** | Proteobacteria | Alphaproteobacteria | Rhodobacterales | Rhodobacteraceae | *Paracoccus* |
|  | **OTU_41** | Proteobacteria | Gammaproteobacteria | Unclassified | Unclassified | Unclassified |
|  | **OTU_44** | Bacteroidetes | Unclassified | Unclassified | Unclassified | Unclassified |
|  | **OTU_46** | Chlamydiae | Chlamydiia | Chlamydiales | Simkaniaceae | *Simkania* |
